# Optimizing accelerometer implementation in a gerotherapeutic trial: Feasibility, adherence, and operational insights of ABLE

**DOI:** 10.1371/journal.pdig.0001691

**Published:** 2026-09-25

**Authors:** Jessica K. Lu, Weilan Wang, Yi Ern Chew, Lihuan Guan, Brian K. Kennedy, Andrea B. Maier

**Affiliations:** 1 Healthy Longevity Translational Research Programme, Yong Loo Lin School of Medicine, National University of Singapore, Singapore, Singapore; 2 NUS Academy for Healthy Longevity, Yong Loo Lin School of Medicine, National University of Singapore, Singapore, Singapore; 3 Centre for Healthy Longevity, National University Health System (NUHS), Singapore, Singapore; 4 Department of Biochemistry, Yong Loo Lin School of Medicine, National University of Singapore, Singapore, Singapore; 5 Department of Human Movement Sciences, @AgeAmsterdam, Faculty of Behavioural and Movement Sciences, Vrije Universiteit Amsterdam, Amsterdam Movement Sciences, Amsterdam, the Netherlands; Lancaster University Medical School, UNITED KINGDOM OF GREAT BRITAIN AND NORTHERN IRELAND

## Abstract

The use of wearable technology for digital phenotyping has been limited in gerotherapeutic trials. Thus, it is important to identify insights into the operational feasibility of adopting accelerometers for digital phenotyping in gerotherapeutic trials, participant adherence to device and intervention, and study dropout risk, particularly in middle-aged adults. This exploratory analysis utilized data from 120 generally healthy, middle-aged adults participating in the Alpha-ketoglutarate (AKG) supplementation and BiologicaL agE in middle-aged adults (ABLE) double-blind, randomized, placebo-controlled trial (ClinicalTrials.gov identifier: NCT05706389). Participants were instructed to wear an ActiGraph wGT3X-BT accelerometer for seven days at four study visits, adhering to a six-month 1 g/day oral AKG supplement regimen with a three-month follow up. Device operational concerns, accelerometer wear behaviour, device wear and supplement use adherence, and study dropouts were evaluated. Associations between device wear (time or adherence) and dropout were analysed using Cox proportional hazards models using study visit as the time scale to account for when participants dropped out from the study. Participants (mean age 50.6 ± 6.0 years, 50% female, n = 59 AKG, n = 61 placebo) showed high adherence: median accelerometer wear was 24 hours per day, > 80% achieved ≥ 4 valid days per visit, and supplement use adherence >90%, with minimal device-related operational concerns across all four visits. Overall study dropout was 17%. In Cox models across study visits, one minute higher in wearing time (hazard ratio (HR): 0.998; 95% confidence interval (CI): 0.997–0.999) and being male (HR: 0.208; CI: 0.065–0.663) were significantly associated with lower dropout risk. Accelerometer deployment proved highly feasible in this gerotherapeutic trial with middle-aged adults. Higher device wearing time and being male were significantly associated with lower study dropout, informing strategies to enhance future trial success.

## Author summary

In our research, we set out to understand how practical it is to use wearable motion sensors to track health patterns in studies focused on healthy aging. While these digital tools offer a wealth of information, we haven’t yet seen them widely used in trials testing treatments that target the aging process. We followed 120 healthy middle-aged adults who were taking a daily supplement called alpha-ketoglutarate, which is being studied for its potential to lower biological age. Over nine months, we asked participants to wear a small activity tracker for one week each at four timepoints. We found that using these devices was highly successful: participants wore them nearly 24 hours a day and remained very consistent with their supplement routine. Our analysis also revealed that people who wore their trackers for longer periods, as well as male participants, were less likely to drop out of the study. This is a vital insight because keeping participants engaged is one of the biggest hurdles in medical research. By proving that this technology is both reliable and well-accepted, our work shows that digital tracking can provide a more continuous, “real-world” look at how new treatments affect us, helping us design more effective longevity interventional trials in the future.

## Introduction

Participant behaviour and adherence to both the intervention and any monitoring protocols are critical for clinical trial success and data integrity [1,2]. Gerotherapeutic trials, which assess interventions targeting biological aging, pose particular challenges for sustained engagement over many months [3]. Although these studies often attract highly motivated, health-conscious individuals [4], real-world adherence tends to diminish once participants leave clinical settings. Digital phenotyping tools (e.g., smart sensors, accelerometers, and wearable devices) enable continuous, objective measurement of both device wear adherence and free-living activity [5,6]. Moreover, by capturing candidate digital biomarkers of aging, these devices can help reveal early signs of intervention responsiveness that traditional endpoints miss [7].

Long-term adherence to interventions and intensive data collection protocols can be difficult [8], especially for middle-aged adults balancing work and family demands [9,10]. Unlike shorter-term exercise and lifestyle studies, gerotherapeutic trials often involve unique interventions (e.g., dietary supplements, repurposed drugs) [11], span over many months [8], and increasingly incorporate objective monitoring tools via digital phenotyping.[7] Although adherence [12] and dropout have been studied extensively in exercise and lifestyle interventions [13,14], detailed operational reports in gerotherapeutic trials are scarce. In particular, few studies describe the feasibility of implementing wearable devices, quantify participant adherence, or examine the associations between adherence and study dropout. Moreover, previous findings may not directly translate to operational feasibility and participant adherence in gerotherapeutic trials, especially among busy middle-aged adults [7].

The aim of this study is to investigate the operational aspects of deploying accelerometers, participant adherence to the accelerometer protocol and supplement use, and associations between accelerometer wear adherence and study dropout in the Alpha-ketoglutarate (AKG) supplementation and BiologicaL agE in middle-aged adults (ABLE) study [15].

## Methods

### Ethics statement

The study was approved by the National University of Singapore Institutional Review Board (NUS-IRB Ref: 2021–946) with all ethical guidelines adhered to under the Declaration of Helsinki [16]. This study was registered in ClinicalTrials.gov with the identifier NCT05706389 (https://clinicaltrials.gov/study/NCT05706389) on January 31, 2023. Written informed consent was obtained from all participants before screening commenced.

## Study design

ABLE is a single-site, parallel, double-blinded, randomized, placebo-controlled trial of community-dwelling middle-aged, healthy adults conducted at Alexandra Hospital (Singapore). Participants were recruited from residents in Singapore to investigate if one gram per day of AKG supplement compared to a placebo could lower DNA methylation age in middle-aged healthy adults. More details on the study protocol were presented in a prior publication [15]. In brief, eligible participants were healthy individuals between the ages of 40 and 60 years of age who were biologically older as defined by DNA methylation age during screening.^15^ Participants invited to the study were randomized (permuted blocks with concealed allocation) at baseline to receive AKG or placebo and scheduled for four study visits (baseline, mid-intervention at three months, end of intervention at six months, and follow-up at nine months). At each visit, participants underwent comprehensive assessments, including biosample collection, anthropometry, questionnaires, and objective activity monitoring, detailed elsewhere.^15^ A total of 120 participants were included in the study from May 4, 2023, to December 19, 2024.

### Data collection

Age, sex, ethnicity, number of diseases, and number of concomitant medications were recorded during screening via a participant survey. Biological age was calculated using the mean of four blood-based principle-component [17] DNA methylation aging clocks, Horvath [18], Hannum [19], PhenoAge [20], and GrimAge [21], detailed elsewhere [15]. Standing height was assessed (to the nearest 0.1 cm) using a stadiometer (SECA217, SECA Gmbh&Co KG, Germany) and weight was assessed (to the nearest 0.1 kg) using a floor weighing scale (SECA813, SECA Gmbh&Co KG, Germany). Body mass index (BMI) was calculated by dividing body mass (kg) by height (m) squared and expressed in kg/m^2^. Waist and hip circumferences were measured (to the nearest 0.1 cm) three times each using a cloth measuring tape (SECA201, SECA Gmbh&Co KG, Germany) at the level of the umbilicus (waist) and at the level of the symphysis pubis and the greatest protrusion of the gluteal muscle (hips), respectively.^15^ Waist-to-hip ratio was calculated by dividing waist circumference by hip circumference.

### Accelerometer protocol and data collection

The ActiGraph wGT3X-BT triaxial accelerometer (firmware Version 1.9.2) (Ametris, LLC, Florida, USA) [22] was used to monitor physical activity (PA) and sedentary behaviour (SB) for seven consecutive days [23–25] from each visit. The accelerometer (weighing 19 g, L×W×T=46×33×15 mm) recorded raw acceleration of movement in three axes and provided raw data expressed in gravitational equivalent units (*g*, for which $\text{1}\;g=\text{9}.\text{8}\text{1}\;m/s^{\text{2}}$) with a range of ±8 g [26]. Data was collected at a samplin*g* frequency of 30 Hz [27], processed using the normal filter, and aggregated over 60-second epochs to detect locomotion and activity based on the orientation and movement of the wrist.^22^ Participants were asked to wear the accelerometer on the non-dominant wrist continuously (while awake and during sleep) for seven days to monitor their routine activity and to remove it only when engaging in water-based activities (such as showering, bathing, or swimming). The accelerometer was given to the participant to wear during each study visit (“Day 0”) between the hours of 8:00 and 11:00 AM (depending on what time they were scheduled to start the visit) followed by an in-person debrief by study staff with the participant at the end of the visit to review device wearing and return instructions, including a hardcopy set of instructions provided in the take-home pack. The hardcopy included instructions to wear the accelerometer consistently, removing it only when participating in water activities, to stick to regular lifestyle routines, record the date and time each time the device was removed and worn again, and inform the study staff if any discomfort was experienced while wearing the device or any other concerns arise (e.g., red light indicating low battery, flashing green light indicating that data recording has not yet begun, etc.). Continuing data collection for seven more days following the visit, participants were instructed to mail back the device to the study team on the eighth day (“Day 8”) in a provided envelope (smartpac, which is a postage-paid, tracked packaging service offered by Singapore Post (SingPost)) with a sticker label of clinic’s address at Alexandra Hospital. The pre-paid envelope contains a bubble-wrap layer the same size as the envelope to protect the device during mailing, and participants can drop off the package at any SingPost posting box, including those at post offices, POPStop counters, POPDrop kiosks, or regular street posting boxes. Once received, study staff removed the woven nylon wristband from the accelerometer and washed it with soap, a viral cleanser, and a bacterial cleanser, then laid it flat to dry. The accelerometer was wiped down with an alcohol wipe. Questions and concerns from participants during the study were answered by the study staff via a dedicated conversation chat on WhatsApp or via email. Device malfunction concerns during the data collection period would only be uncovered following the return of the device in the mail and data was exported. There were no reminder messages from the study staff to actively encourage participants to wear the device during the monitoring period. The ActiGraph device has no buttons or display and can operate for around 25 days on a full charge without any user interaction from the participant (low user burden). Participants were eligible to refuse wearing the device while still participating in the clinical trial. Device malfunctions, strap concerns, data loss incidents, and participant-reported concerns were recorded by study staff. The participants were asked to record daily in the electronic PA diary the exercises that they engage in and their monitor removal times (and why it was removed, e.g., shower, swimming, etc.). When sleep/wake times were missing, study staff estimated sleep and wake periods using the accelerometer movement pattern together with available diary entries for adjacent days. Diary-recorded device removal periods were cross-checked against ActiGraph-detected non-wear periods. Where diary entries and accelerometer signals were discrepant, accelerometer-derived non-wear detection was prioritized for wear-time calculations.

### Accelerometer data processing and quality control

Accelerometer files (.gt3x) were downloaded from the device using ActiSync (syncing application to communicate between ActiGraph devices and CentrePoint, software Version 3.17.0 to 3.23.0). The raw data from the device was then uploaded to the manufacturer’s cloud-based software platform (https://studyadmin.actigraphcorp.com/). The CentrePoint cloud service (CentrePoint software Version 4.2.1.1 to 5.1.0.2, ActiGraph, LLC, USA) provided by the manufacturer was used to obtain detailed reports of device wear time [28]. Files were analyzed in 60-second epochs and wear time was validated using the wear time validation tool in the CentrePoint software to detect periods of accelerometer non-wear time [29,30]. Non-wear time was defined as periods of ≥90 consecutive minutes of low acceleration (zero vector magnitude counts, allowing for ≤2 minutes of non-zero counts, with small window length of 30 min) with little variability and excluded from the analyses (vector magnitude enabled, meaning the vector magnitude of three-axis data is used when available, otherwise only axis 1 is used) [31,32]. These definitions have been widely used in previous studies with accelerometers [33,34]. The device idle sleep mode was enabled (i.e., the device checks once every second to determine if the unit has moved. If there is movement, the device exits sleep mode to continue sampling) [35]. The high-resolution raw comma-separated values (CSV) files with minute-by-minute data from the ActiGraph device was exported for further analysis.^28^ Following standard 24-h accelerometer protocols, a valid day was defined as at least 16 hours (≥960 minutes) of wear within a 24-h period) [36–38]. Participants were included in the analyses if they recorded at least one valid day of accelerometer data between the instructed Day 1 to Day 7, inclusive, at each visit.

### Participant adherence

Participant adherence was assessed in two domains: device wear adherence and supplement use adherence. Adherence was verified using total wear minutes per day, extracted from the exported CSV files. Individual device adherence was defined as the proportion of days the participant wore the accelerometer for at least 16 hours (960 minutes). Non-adherent days were defined as days with less than 16 hours of wear. Thus,


$$\begin{array}{c} Individual\;device\;wear\;adherence\;(\%)=\left(\frac{Days\;with\;valid\;wear\;time}{Total\;monitoring\;days}\right)\times \text{1}\text{0}\text{0}\text{,} \end{array}$$

where total monitoring days refers to the instructed seven days of wear following the study visit. If participants had at least four valid days of data [27], they were considered as adherent for the monitoring period of that visit.

Participants were asked to return their supplement bottles at each follow-up visit, followed by counting of leftover supplement pills by study staff. Supplement use adherence was calculated as the proportion of supplement pills taken relative to the expected dosage:


$$\begin{array}{c} \text{Adherence }(\%)=\left(\frac{Supplement\;pills\;dispensed-Supplement\;pills\;returned}{Supplement\;pills\;dispensed}\right)\times \text{1}\text{0}\text{0}\text{.} \end{array}$$

Supplement pills dispensed refers to the number of supplement pills provided to the participant at the baseline and mid-intervention visits, while supplement pills returned refers to the number of unconsumed supplement pills returned at the end of intervention and follow-up visits. The expected dosage was 360 supplement pills (two pills per day for the six-month supplementation period). Additionally, proportion of days covered (PDC) was used as a more objective metric of adherence, accounting for the number of days the participant had the supplement on hand. Thus,


$$\begin{array}{c} PDC=\left(\frac{Number\;of\;days\;covered\;by\;the\;supplement\;supply}{Total\;days\;in\;measurement\;period}\right)\times \text{1}\text{0}\text{0}\text{,\textasciitilde{}and} \end{array}$$

$$\begin{array}{c} Supplement\;available=\left(\frac{\left(Pills\;dispensed)-(Pillls\;returned\right)}{Daily\;dose\;(\text{2}\;pills/day)}\right)\text{,} \end{array}$$

where the number of days covered represents the days in which the participant had the supplement available and the total days in measurement period corresponds to the number of days between the baseline and end of intervention visits (stop point of supplementation). For participants who had dropped out before the end of intervention, the calculated end date was set as the date of their last visit plus 45 days (midpoint between two visits, which is separated by 90 days) to account for participants who withdrew in between visits.

### Study dropout rates

Study dropout rates were assessed based on participant attendance at the mid-intervention, end of intervention, and follow-up visits. Dropout was defined as any participant who did not attend a follow-up visit after the baseline visit or formally withdrew from the study. Thus,


$$\begin{array}{c} Study\;dropout\;rate=\left(\frac{Number\;of\;participants\;who\;dropped\;out\;after\;the\;baseline\;visit}{Total\;enrolled\;participants}\right)\times \text{1}\text{0}\text{0}\text{.} \end{array}$$

Additionally, time-to-dropout was assessed by categorizing participants based on whether they dropped out after visits at baseline, mid-intervention, or end of intervention, or if they completed the study. Reasons for dropout were recorded if participants provided them (voluntarily).

### Statistical analyses

Descriptive statistics for continuous variables with a normal distribution were presented as mean ± standard deviation (SD) and those with a non-normal distribution as median [interquartile range, IQR]. Categorical variables were presented as frequency (percentage). Numerical variables were compared using independent or Welch two sample *t*-tests (normal distribution) and the Wilcoxon rank sum tests (non-normal distribution). If there is statistical significance, the rank biserial correlation (*r*) for non-normally distributed variables was calculated to find the effect size. The effect size for a value close to 0 suggests negligible associations, 0.10–0.29 is considered small, 0.30–0.49 is medium, and 0.50 and above is large [39]. Categorical variables were compared using $\chi^{\text{2}}$-tests or Fisher’s exact tests.

Day-to-day variability in wear time was calculated for each participant per monitoring period and summarized across participants as a measure of wear consistency and reliability. It was calculated using the coefficient of variation (CV):


$$\begin{array}{c} Coefficient\;of\;variation\;(CV)=\left(\frac{standard\;deviation\;of\;wearing\;time}{mean\;wearing\;time\;across\;the\;seven\;days}\times \text{1}\text{0}\text{0}\right)\text{.} \end{array}$$

A lower coefficient of variation percentage indicates lower variability and, therefore, higher regularity of day-to-day wearing time [40].

Time-to-dropout was analysed using Kaplan-Meier time-to-event analysis and Cox proportional hazards models to identify factors associated with dropout risk over the course of the study. Study visit was used as the time scale, with dropout defined as the first missed follow-up visit or formal withdrawal after baseline. Device wear time and device wear adherence were modelled as visit-level time-varying covariates. For each risk interval, wear data from the preceding monitoring period were aligned with subsequent dropout status, such as accelerometer wear after a completed visit was used to model dropout before the next scheduled visit. Cox models used cluster-robust standard errors clustered by participant to account for repeated visit-level observations. Candidate time-varying covariates, including device wear time and device wear adherence, were screened in univariate models. Because device wear time and wear adherence were highly collinear, the variable with the superior concordance index in univariate screening was chosen for multivariable analysis. The adjusted Cox model included device wear time, age, sex, and intervention or placebo group covariates and accounted for the time to dropout. The proportional hazards assumption was assessed using Schoenfeld residuals. Where evidence of non-proportionality was detected for adjustment covariates, sensitivity analyses were conducted to evaluate whether the main associations for device wear time and sex were robust. Logistic regression was used as a sensitivity analysis to assess associations of device wear behaviour with overall study dropout.

The statistical significance level was set at p<0.05, and a trend was defined as a p value of 0.05≥p<0.10. Data was analyzed using statistical analysis software (R version 4.2.2, RStudio Version 2024.09.0 + 375). Library packages lme4 (Version 1.1.36) and survminer (Version 0.5.0) were used.

### Role of funding source

The funders of the study had no role in study design, data collection, data analysis, data interpretation, writing of the report, or the decision to submit for publication.

## Results

**Fig 1:**
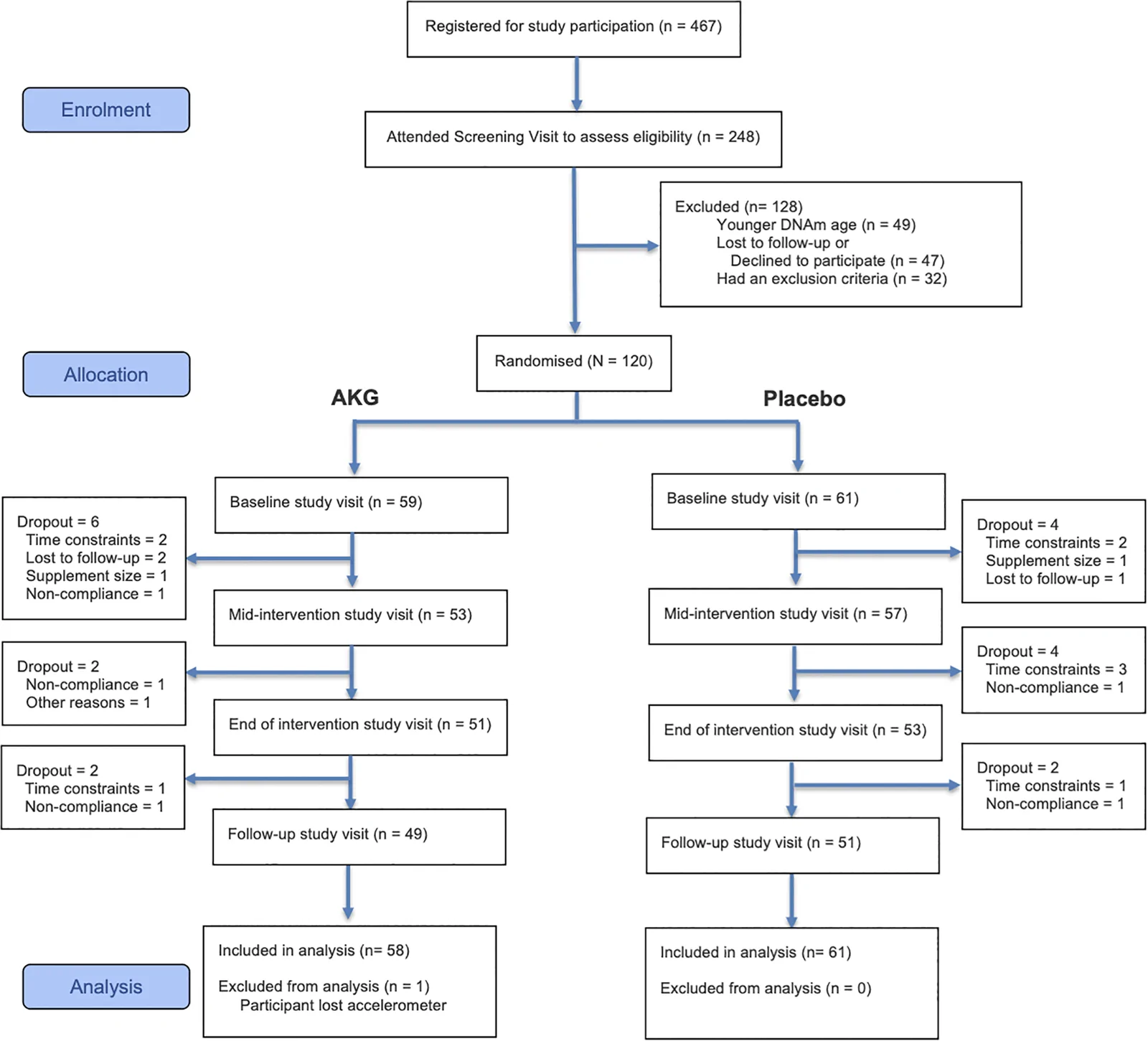
CONSORT Flow Diagram of the ABLE study. Flow diagram of the progress through the phases of the ABLE randomised controlled trial of two groups (that is, enrolment, intervention allocation, follow-up, and data analysis). AKG, alpha-ketoglutarate intervention group; DNAm age, DNA methylation age; Placebo, control group.

A flow of participants throughout the study is presented in Fig 1. The baseline characteristics of the 120 participants (AKG: n = 59, placebo: n = 61) with a mean ± SD chronological age of 51.1 ± 6.2 (AKG) and 50.5 ± 5.8 (placebo) years are summarized in Table 1. The mean DNA methylation age of the AKG participants (49.2% female) was 55.2 ± 6.5 years and placebo participants (50.8% female) was 53.7 ± 6.0 years. The mean BMI and median waist-to-hip ratio was 22.6 ± 2.6 kg/m^2^ and 0.8 [0.8–0.9] in the AKG group and 21.9 ± 2.7 kg/m^2^ and 0.8 [0.8–0.9] in the placebo group, respectively. No statistically significant differences were observed between the AKG intervention and placebo groups at baseline (Table 1).

**Table 1:** Characteristics of ABLE participants at baseline between the intervention and placebo groups.

	AKG (*n* = 59)	Placebo (*n* = 61)	*p*
Age, years	51.1 ± 6.2	50.5 ± 5.8	0.542
DNA methylation age, years	55.2 ± 6.5	53.7 ± 6.0	0.170
Female, n (%)	29 (49.2)	31 (50.8)	1.000
Ethnicity, n (%)	59 (49.2)	61 (50.8)	0.811
Chinese	51 (86.4)	56 (91.8)	
Malay	2 (3.4)	1 (1.6)	
Indian	2 (3.4)	1 (1.6)	
Caucasian	4 (6.8)	3 (4.9)	
Medical history (diseases), n (%)	51 (86)	60 (98)	
Musculoskeletal and tegmental	10 (17)	10 (16)	1.000
Haematologic	8 (14)	9 (15)	1.000
Ophthalmologic	8 (14)	8 (13)	1.000
Others	25 (42)	33 (54)	0.165
Number of medications	0 [0–0]	0 [0–0]	0.984
Height, cm	164.8 ± 8.1	166.2 ± 8.4	0.348
Weight, kg	60.3 [53.7–67.7]	60.5 [52.1–67.9]	0.838
BMI, kg/m^2^	22.6 ± 2.6	21.9 ± 2.7	0.147
Waist circumference, cm	75.7 [71.5–81.2]	76.7 [69.3–81.2]	0.871
Hip circumference, cm	94.4 [91.1–97.0]	92.3 [90.3–97.5]	0.536
Waist-to-hip ratio	0.8 [0.8–0.9]	0.8 [0.8–0.9]	0.969

Date are presented as mean ± standard deviation (SD) or median [interquartile range (IQR)] unless otherwise stated.

Medical history quantifies the number of participants with at least one disease in each category, and participants can have diseases in more than one category.

*p*-value refers to the comparison between the AKG intervention and placebo groups.

Abbreviations: AKG, alpha-ketoglutarate intervention group; BMI, Body Mass Index; bpm, beats per minute; cm, centimetre; kg, kilogram; kg/m^2^, kilograms per square metre.

All 120 randomized participants agreed to wear the device at the baseline visit. One participant lost the device during the baseline visit and withdrew after the baseline visit, resulting in accelerometer data captured from 119 participants. A total of six participants reported specific concerns related to accelerometer wear, impacting eight participant-visits (Table 2). These included one participant (0.8% of all participants) who refused to wear the device due to its appearance, two instances of device loss (1.7%), and three participants (2.5%) who reported distinct comfort or strap-related concerns (skin irritation, skin rash, and strap hygiene). The participant refused to wear the device due to its aesthetics following the mid-intervention, end of intervention, and follow-up visits. Strap-related discomfort led to a strap change for one participant, while another reported a rash. Overall, 5% of participants reported specific device-related concerns impacting 8 out of 432 participant-visits.

**Table 2:** Participant-reported accelerometer concerns, including reasons for non-wear or refusal.

Reason category	n of all participants	Specific detail	n of all visits affected
Refusal	1	Aesthetics; disliked appearance, “looks like a prison tracker”	3
Device loss	2	Device was lost by participant	2
Comfort/wrist strap: skin irritation	1	Reported strap caused skin irritation (strap changed within monitoring period)	1
Comfort/wrist strap: skin rash	1	Reported developed rash from strap	1
Comfort/wrist strap: hygiene	1	Commented strap from previous monitoring period/visit became smelly when wet	1
**Total participants**	**6**	**Total visits affected**	**8**

Note: skin irritation refers to participant informing study team of irritation felt on the skin, but there was no visible symptom; skin rash refers to participant informing study team of rash developing on the skin (visible symptom).

n of all participants: unique participants experiencing this specific concern

n of all visits affected: the quantified impact of that specific concern across monitoring periods

Across the instructed seven days of monitoring, wearing time was highest between Day 2 and Day 6, inclusive (S1 Fig). The median [IQR] of the daily device wearing time across all visits was 1400 [1245–1440] min (S2 Fig). The median wear time during weekdays was 1340 [886–1440] min and 1440 [1063–1440] min during weekends (*p* < 0.001) (S3 Fig) with a small rank biserial correlation effect size of *r* = 0.105. At baseline, the AKG group recorded a median device wearing time of 1440 [972–1440] min compared to 1440 [920–1440] min in the placebo group (*p* < 0.001) (Table 3) with a small effect size *r* = 0.102. There was a statistical difference between the AKG and placebo groups in valid days captured at baseline and at end of intervention, even though the median valid days recorded for both groups was 7 [7–7] days at baseline (*p* = 0.026) and 7 [7–7] and 7 [6–7] days for the AKG and placebo groups, respectively, at the end of intervention (*p* = 0.001) (Table 3). The effect size of the difference in valid days was *r* = 0.082 at baseline and *r* = 0.134 at end of intervention. There was no difference in wearing time between groups at the mid-intervention, end of intervention, and follow-up visits or in the variability of wearing time.

**Table 3:** Accelerometer device wearing adherence of participants at study visits between the intervention and placebo groups.

	Baseline (*n* = 119)			Mid-intervention (*n* = 110)			End of intervention (*n* = 102)			Follow-up (*n* = 98)		
	AKG (*n* = 58)	Placebo (*n* = 61)	*p*	AKG (*n* = 53)	Placebo (*n* = 57)	*p*	AKG (*n* = 51)	Placebo (*n* = 51)	*p*	AKG (*n* = 48)	Placebo (*n* = 50)	*p*
Wearing time, min/day	1440 [972–1440]	1440 [920–1440]	**<0.001**	1326 [884–1440]	1323 [812–1440]	0.445	1440 [910–1440]	1348 [863–1440]	0.398	1345 [910–1440]	1321 [866–1440]	0.103
CV of wearing time, %	7.5 ± 9.8	17.1 ± 35.2	0.109	24.3 ± 47.5	31.6 ± 55.8	0.815	23.4 ± 46.8	22.2 ± 46.8	0.602	18.0 ± 19.3	20.9 ± 28.9	0.989
Valid days, days	7 [7–7]	7 [7–7]	**0.026**	7 [6–7]	7 [6–7]	0.169	7 [7–7]	7 [6–7]	**0.001**	7 [6–7]	7 [6–7]	0.664
Individual device adherence, %	100 [100–100]	100 [86–100]	0.191	86 [71–100]	100 [71–100]	0.856	100 [86–100]	100 [71–100]	0.416	100 [82–100]	100 [75–100]	0.642
Adherent, n (%)	56 (97)	55 (90)	0.924	43 (81)	46 (81)	0.751	42 (82)	45 (88)	0.748	41 (85)	41 (82)	1.000

Data are presented as mean ± standard deviation (SD) or median [interquartile range (IQR)] unless otherwise stated.

Wearing time includes all instructed monitoring days (Day 1 to Day 7). Valid days refers to how many instructed monitoring days (Day 1 to Day 7) had wearing time of at least 16 hours (≥960 mins). Individual device adherence refers to the percentage of days out of the seven instructed monitoring days had wearing time ≥16 hours. Adherent refers to the proportion of participants who had greater than four valid days out of the seven instructed days of monitoring.

*P*-value refers to the comparison between the AKG intervention and placebo groups; *p* < 0.050 is presented in **bold**.

Abbreviations: AKG, alpha-ketoglutarate intervention group; CV, coefficient of variation.

**Fig 2:**
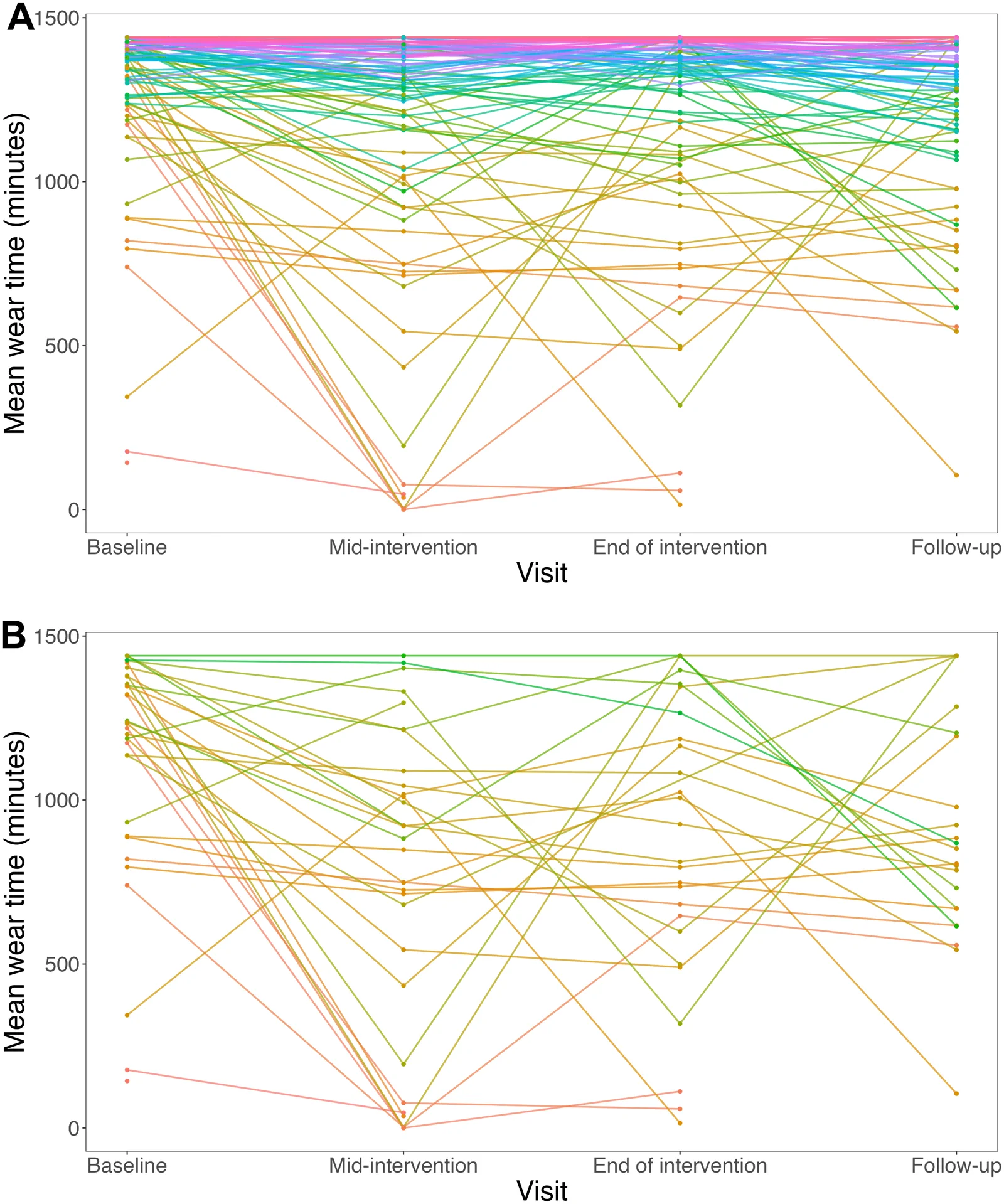
Mean accelerometer device wear time for each visit across all four clinical study visits for each participant. (A) All participants (N = 119). (B) Participants with <960 minutes of mean wear time at one or more than one visit (n = 33). Each line (distinct colour) shows one participant’s daily mean wear time (Days 1–7) at each of the four visits, ordered top-to-bottom by descending average wear time. Colours remain consistent across visits; lines terminate when a participant withdrew or did not wear the device in a subsequent visit.

**Fig 3:**
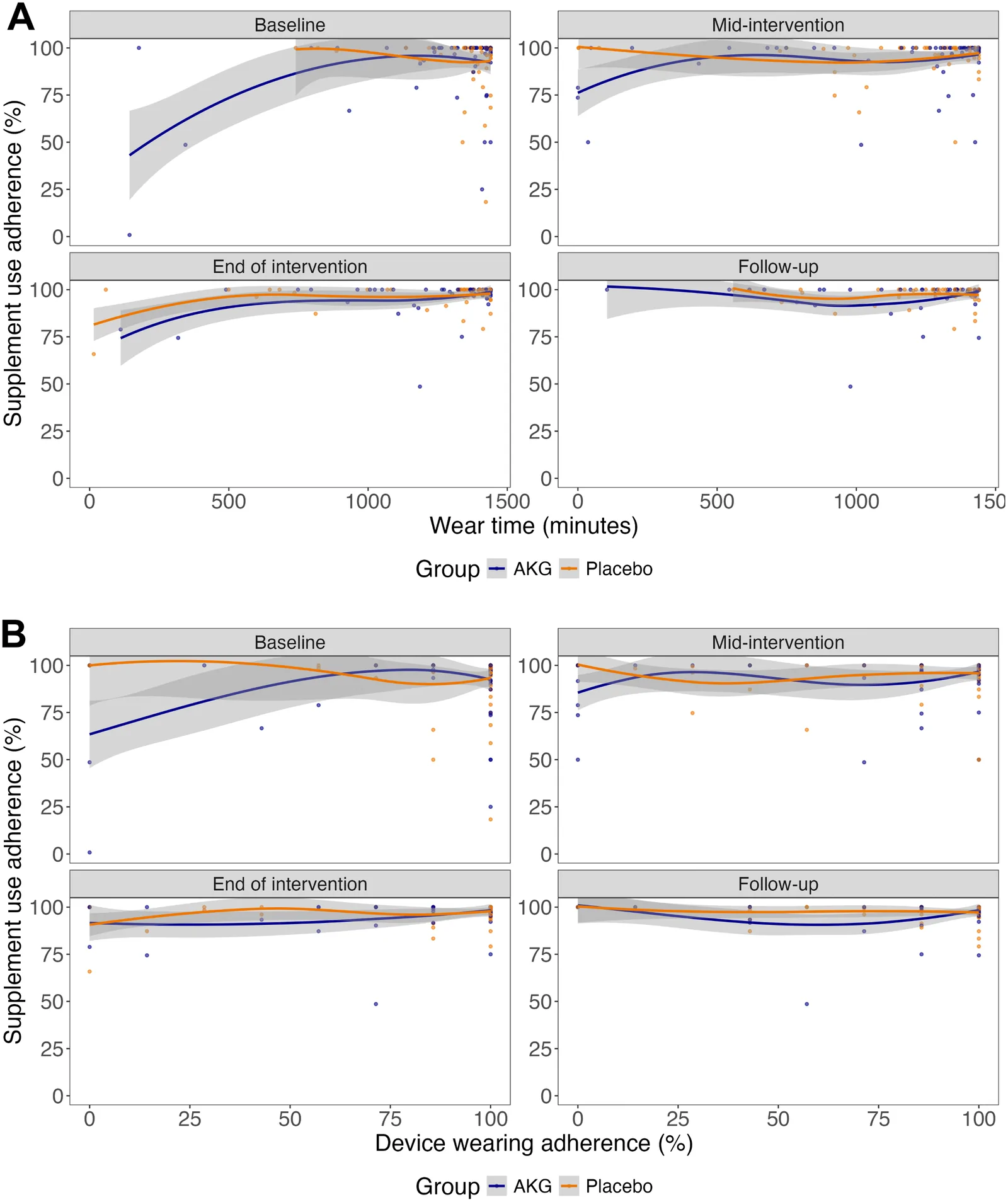
The association between supplement use adherence and wear time (A) or device wearing adherence (B) across the four visits by the intervention or placebo group. There were N = 119, 110, 102, and 98 participants overall at baseline, mid-intervention, end-of-intervention, and follow-up, respectively. Each dot represents the supplement use adherence against mean device wear time (minutes, **A**) or device wearing adherence (%, **B**) for each participant at each respective visit. The trend line in each plot is the locally estimated scatterplot smoothing (non-parametric method that fits multiple regression) with the standard error in shaded grey at each visit for the intervention (AKG) and placebo groups.

Adherence was assessed for all 120 participants across the four visits, including device wear adherence (n = 119) and supplement use adherence (n = 120), accounting for time to dropout. Participants with higher adherence in wearing the device after the baseline visit had higher adherence across all four visits (Fig 2A and 2B). The median individual device wearing adherence and the proportion of participants adherent to wearing the device were both greater than 80% over the course of the study with no significant differences between the two groups (Table 3). The supplement use adherence was 100 [95–100]% for both groups while the PDC was 93 [82–98]% and 95 [86–98]% for the AKG and placebo groups, respectively. Neither device wear time (Fig 3A) nor device wearing adherence (Fig 3B) showed associations with supplement use adherence.

The overall study dropout rate was 17% (20 out of 120 participants dropped out after randomization to the AKG or placebo groups). The primary reasons for withdrawal were work/schedule-related conflicts (n = 10), followed by lost to follow-up or uncontactable (n = 4). Other reasons included study product-related issues (n = 2) and protocol non-compliance (n = 2), as detailed in Table 4. A time-to-event analysis of study dropout showed no significant difference between the groups (log-rank *p* = 0.92), consistent with ~83% completion probability in both groups (S4 Fig). Device wear time had the highest concordance, which means this univariate Cox model discriminated better who had higher versus lower hazards of dropout by correctly ranking participant-pairs using device wear time alone (S1 Table). One minute higher in wearing time was associated with a 0.2% lower hazard risk of dropping out (HR: 0.998; 95% CI: 0.997–0.999; *p* < 0.001) as well as being male (HR: 0.208; 95% CI: 0.065–0.663; *p* = 0.008) in the adjusted multivariate Cox model. Schoenfeld residual testing showed no evidence of non-proportionality for device wear time, sex, or intervention group in the primary adjusted model, although age showed evidence of non-proportionality and the global test was significant. In a sensitivity model excluding age, the proportional hazards assumption was met globally and for all included covariates. Device wear time and sex remained associated with dropout risk, with estimates similar to the primary adjusted model (wear time HR: 0.998, 95% CI: 0.997–0.999, *p* < 0.001; sex HR: 0.198, 95% CI: 0.063–0.624, *p* = 0.006). Consistent with the time-to-event findings, one minute higher in wearing time (OR: 0.998; 95% CI: 0.996–0.999; *p* = 0.029) and being male (OR: 0.174; 95% CI: 0.044–0.545; *p* = 0.005) was associated with lower odds of dropping out overall in the logistic regression model (Table 5).

**Table 4:** Reasons for dropout and withdrawal from the study (total N = 20 dropouts).

Reason category	Participants
Work/schedule-related conflicts	10
Time commitment/overall study burden	1
Lost to follow-up/uncontactable	4
Participant medical reasons (unrelated to study product)	1
Study product-related issues	2
Protocol non-compliance/withdrawal of cooperation	2
**Total participants**	**20**

**Table 5:** The association between device wear time and dropout status in middle-aged, healthy adults in a gerotherapeutic trial (N = 119).

	Time-to-event hazard of dropping out			Overall odds of dropping out		
	HR	95% CI	*P*	OR	95% CI	*P*
Wear time, min/day	0.998	0.997–0.999	**<0.001**	0.998	0.996–0.999	**0.029**
Age, years	0.954	0.882–1.032	0.242	—	—	—
Sex (male)	0.208	0.065–0.663	**0.008**	0.174	0.044–0.545	**0.005**
Group (AKG)	1.207	0.463–3.142	0.701	—	—	—

Multivariate cox proportional-hazards models for risk of dropout.

Hazard ratios (HR) < 1 indicate a lower instantaneous risk of dropping out; HR > 1 indicate a higher risk.

Interpretation for Cox regression: One minute per day higher in wearing time was associated with a lower hazard of study dropout risk.

Multivariate logistic regression model for risk of dropout.

Odds ratio (OR) < 1 indicate lower odds of dropping out; OR > 1 indicate higher odds.

Interpretation of logistic regression: One minute higher in wearing time was associated with lower odds of study dropout risk.

Adjusted models: age, sex, and intervention or placebo group adjusted.

*P < 0.050* presented in **bold**.

Abbreviations: AKG, intervention group; CI, confidence interval; HR, hazards ratio; min, minutes; OR, odds ratio.

1. Work/Schedule-Related Conflicts: Participant explicitly states work commitments, new job, inability to take leave, or other schedule incompatibilities (e.g., extended travel/holiday precluding visits) as the primary reason for not being able to meet study requirements.
2. Time Commitment/Overall Study Burden: Participant finds the overall demands of the study (visits, procedures like blood draws, tracker use, supplement intake) too high or underestimated the commitment, leading to withdrawal.
3. Participant Medical Reasons (Unrelated to Study Product): Withdrawal due to new or existing health issues, accidents, or sickness not considered related to the investigational product.
4. Study Product-Related Issues: Withdrawal due to issues with the study supplement itself, such as difficulty swallowing, perceived side effects, or adverse events attributed to the product.
5. Lost to Follow-up/Uncontactable: Participant could not be reached for follow-up visits despite multiple attempts.
6. Protocol Non-Compliance/Withdrawal of Cooperation: Participant actively refuses to adhere to key study protocols, is unwilling to continue with assessments, or expresses a general unwillingness to commit further for reasons not fitting other categories. This includes challenging interactions or explicit refusal to cooperate.

## Discussion

This study demonstrates the implementation of accelerometry in a nine-month gerotherapeutic trial involving community-dwelling, biologically older yet generally healthy middle-aged adults. There were minimal device-related operational concerns reported, and the adherence to wearing the device and supplement use were both high. Higher device wear time and being male were significantly associated with lower risk of study dropout.

Operational aspects of device deployment were generally manageable as only a small fraction of participants reported specific device-related concerns. Outright refusal due to aesthetics was minimal, underscoring general acceptability of wearing the ActiGraph wGT3X-BT accelerometer in this gerotherapeutic trial. Device loss represents a practical challenge leading to data loss. For future trials, strategies like reminders to participants about device usage, use of devices with remote data upload capabilities, and immediate data retrieval post-wear period could mitigate such data loss [41]. The current protocol, requiring the device to be mailed back before data retrieval, inherently carries this risk. Comfort and strap-related concerns, including skin irritation, rash, and hygiene concerns, highlight the importance of material selection and participant support.^41^ Promptly addressing these by changing straps or improving sanitisation protocols following device return appeared effective and underscores the need for proactive management and comfortable, hypoallergenic materials in wearable device selection.^41^

The observed increase in wear time immediately post-visit followed by stabilization and a slight decline before device return is consistent with patterns seen in other studies and may reflect an initial Hawthorne effect or heightened awareness [42], normalizing over the wear period. The variability in wear time, which was less than 35% in this study, seems to be low, indicating reliability in data captured. While a study in middle-aged Australian adults reported day-to-day variability in physical activity [40], they did not report on the variability in device wearing time; thus, it was difficult to determine whether this variability is considered low. One of the key findings, however, for the feasibility of incorporating accelerometers in this demographic and trial type was the high device wearing adherence observed. While the current adherence rates were high, there are no similar longitudinal studies in generally healthy middle-aged populations reporting device wear adherence. The most comparable population is a cohort study in adults from the USA between 18–74 years, of whom 78% were adherent with at least one day of data [43]. A systematic review also reported overall device adherence of 59% at last follow-up in adult populations with cardiovascular disease [44]. Overall, despite being a typically busy demographic (middle-aged adults balancing work and family) [9,10], participants may have been more motivated due to the nature of voluntarily participating in a gerotherapeutic trial, which was well-supported by study staff, and found the standard seven-day wear period manageable [25]. Participants also demonstrated high adherence to the investigational product, with median supplement use adherence exceeding 90%. This high adherence to supplement use over six months is noteworthy, where adherence in control subjects had been 77% and 84% in treatment subjects across 771 adherence intervention studies [45]. However, device wear adherence was not associated with supplement use adherence, indicating there may be other factors leading to high supplement use adherence, such as motivation [46] and product tolerance [47].

The overall study dropout rate of 17% is comparable to rates of 20% reported in similar duration clinical trials involving middle-aged adults [48]. The primary reasons for withdrawal were dominated by work/schedule-related conflicts, a common challenge in studies with this demographic, followed by lost to follow-up. Another key finding was the association between device wear time and study dropout risk. Higher wearing time and being male were significantly associated with lower odds of dropout. This suggests that adherence to wearing the device could serve as a proxy for overall study engagement, commitment, or perhaps perceived benefit, which in turn influences participant retention. This finding warrants further exploration, potentially relating to differing motivations, health perceptions, or tolerance for study burden between sexes in this cohort [49]. One plausible explanation is that some female participants may have experienced higher perceived study burden because of competing work, family, and caregiving responsibilities, which have been identified as barriers to clinical trial participation in prior research [49,50]. However, the present study did not measure caregiving responsibilities or perceived study burden. These mechanisms should, therefore, be considered hypothesis-generating and can be assessed in future gerotherapeutic trials. The lack of significant differences in adherence (device or supplement use) and dropout rates between the AKG and placebo groups suggest the intervention itself did not overtly impact these feasibility or dropout risk outcomes.

This study provides several actionable insights for future trials, particularly gerotherapeutic trials, incorporating accelerometers in middle-aged community-dwelling adults, such as participant onboarding and support, device management and data retrieval, optimizing wear protocols, and data quality and quality control [41], all of which aid in achieving a high data yield. Clear instructions, accessible troubleshooting (e.g., WhatsApp support), and managing expectations regarding device wear (including aesthetics and comfort) are crucial. Proactive strap replacement or material options can mitigate comfort concerns. Strategies to minimize data loss from lost devices are also needed. While mail-back is logistically simple, future studies could explore devices with periodic or near real-time data syncing capabilities, especially for longer wear periods or more critical data endpoints [51]. A standard seven-day wear protocol [34] post-visit appears manageable for this population. Moreover, defining clear non-wear criteria, valid day definitions, and monitoring wear time variability can ensure data reliability. The consistent individual adherence patterns across visits observed suggest that early compliance may lead to later device adherence, allowing for targeted support. The combination of these strategies may have contributed to a higher proportion of participants providing sufficient valid days for PA analysis. Given that being male was associated with lower dropout risk in this analysis, future studies should consider whether additional retention support is needed for female participants or other groups with competing work or caregiving demands [52]. Such support should be designed to reduce burden rather than to assume non-adherence, and future trials should directly measure potential mediators such as caregiving responsibilities, work constraints, perceived burden, and motivation [53].

One of the strengths of this study included using an objective measurement of PA and wear time with longitudinal data across four visits. Another strength is detailed operational tracking and records of issues and concerns raised by participants. This is also one of the first studies to focus on objectively measured PA and SB in a biologically older yet relatively healthy middle-aged cohort in a gerotherapeutic trial setting. A possible limitation is that the single-center design may limit generalizability. The specific ActiGraph model used also has its own characteristics [22], and findings could differ with other devices. Increasing the period of monitoring from each visit may also provide a more complete picture of device adherence in a gerotherapeutic study [54]. Reasons for non-adherence could also be explored in future analyses for a better understanding of participant behaviour and engagement in gerotherapeutic trials.

## Conclusion

The current findings underscore that accelerometer integration is feasible and can yield high-quality data in middle-aged adults in a gerotherapeutic interventional trial. Future gerotherapeutic trials should consider study designs for robust participant support and device management. The operational insights offer valuable guidance for optimizing the design and execution of future aging research incorporating wearable technology.

## Supporting information

S1 Table The association between device wear time or wearing adherence and dropout status in generally healthy middle-aged adults in a gerotherapeutic trial (N = 119). (DOCX)

S1 Fig Accelerometer device wearing minutes from visit date (Day 0) to eight days after (Day 8) across all four visits (N = 119). Trend line is the locally estimated scatterplot smoothing (non-parametric method that fits multiple regression) with the standard error in shaded grey for the alpha-ketoglutarate (AKG) intervention (dark blue) and Placebo (orange) groups.
(DOCX)

S2 Fig Wear time distribution of all participant-days across all four visits. The red and green dotted line indicates the 25th percentile (1245 minutes) and 75th percentile (1440 minutes) of the participant-days’ wearing time of the accelerometer. The median daily device wear time across all four visits was 1440 minutes during the instructed Days 1–7 of the monitoring periods. Data from N = 119 participants and N = 432 visits were included.
(DOCX)

S3 Fig Device wear time for Days 1–7 sorted by weekday and weekend days across all visits for each participant expressed as scatterplots and box-and-whiskers plot (N = 119). Each colour indicates a study visit (blue for baseline, orange for mid-intervention, green for end of intervention, and red for follow-up). The scatterplots for each visit are superimposed while the box-and-whiskers plot indicating the 25th percentile, median, and 75th percentile of wear time (minutes) aligned side-by-side for each visit.
(DOCX)

S4 Fig Kaplan-Meier curve for time-to-event analysis of participant dropout from the study. Blue refers to the AKG intervention group and orange refers to the placebo group at each study visit.
(DOCX)
