# Supplementary material for: Optimizing accelerometer implementation in a gerotherapeutic trial: Feasibility, adherence, and operational insights of ABLE
Source: PLOS Digit Health. 2026 Sep 25;5(9):e0001691. doi: 10.1371/journal.pdig.0001691 (PMC13614584; doi:10.1371/journal.pdig.0001691)
Supplement: Supplementary file 1 [file pdig.0001691.s001.docx]

**S1 Table. The association between device wear time or wearing adherence and dropout status in generally healthy middle-aged adults in a gerotherapeutic trial (N = 119).**

|  | Time-to-event hazard of dropping out | | |  |  |
| --- | --- | --- | --- | --- | --- |
|  | HR | 95% CI | *P* | Concordance | Score (logrank) test Robust |
| Wear time, minutes | 0.998 | 0.997–0.999 | **<0.001** | 0.646 | 4.78 |
| Wearing adherence, % | 0.985 | 0.975–0.994 | 0.002 | 0.621 | 4.39 |

Univariate cox proportional-hazards models for time to dropout. Hazard ratios (HR) < 1 indicate a lower instantaneous risk of dropping out; HR > 1 indicate a higher risk.

Example interpretation: One minute higher in wearing time was associated with a 0.3% lower hazard of dropout (HR 0.998; 95% CI 0.997–0.999; p < 0.001).

*p* < 0.001 is presented in **bold**.
