# Supplementary material for: Optimizing accelerometer implementation in a gerotherapeutic trial: Feasibility, adherence, and operational insights of ABLE
Source: PLOS Digit Health. 2026 Sep 25;5(9):e0001691. doi: 10.1371/journal.pdig.0001691 (PMC13614584; doi:10.1371/journal.pdig.0001691)
Supplement: Supplementary file 2 [file pdig.0001691.s002.docx]

**S1 Fig. Accelerometer device wearing minutes from visit date (Day 0) to eight days after (Day 8) across all four visits (N = 119).** Trend line is the locally estimated scatterplot smoothing (non-parametric method that fits multiple regression) with the standard error in shaded grey for the alpha-ketoglutarate (AKG) intervention (dark blue) and Placebo (orange) groups.
