# Supplementary material for: Optimizing accelerometer implementation in a gerotherapeutic trial: Feasibility, adherence, and operational insights of ABLE
Source: PLOS Digit Health. 2026 Sep 25;5(9):e0001691. doi: 10.1371/journal.pdig.0001691 (PMC13614584; doi:10.1371/journal.pdig.0001691)
Supplement: Supplementary file 3 [file pdig.0001691.s003.docx]

**S2 Fig. Wear time distribution of all participant-days across all four visits.** The red and green dotted line indicates the 25^th^ percentile (1245 minutes) and 75^th^ percentile (1440 minutes) of the participant-days’ wearing time of the accelerometer. The median daily device wear time across all four visits was 1440 minutes during the instructed Days 1 to 7 of the monitoring periods. Data from N = 119 participants and N = 432 visits were included.
