# Supplementary material for: Optimizing accelerometer implementation in a gerotherapeutic trial: Feasibility, adherence, and operational insights of ABLE
Source: PLOS Digit Health. 2026 Sep 25;5(9):e0001691. doi: 10.1371/journal.pdig.0001691 (PMC13614584; doi:10.1371/journal.pdig.0001691)
Supplement: Supplementary file 4 [file pdig.0001691.s004.docx]

**S3 Fig. Device wear time for Days 1 to 7 sorted by weekday and weekend days across all visits for each participant expressed as scatterplots and box-and-whiskers plot (N = 119)**. Each colour indicates a study visit (blue for baseline, orange for mid-intervention, green for end of intervention, and red for follow-up). The scatterplots for each visit are superimposed while the box-and-whiskers plot indicating the 25^th^ percentile, median, and 75^th^ percentile of wear time (minutes) aligned side-by-side for each visit.
