# Supplementary material for: Optimizing accelerometer implementation in a gerotherapeutic trial: Feasibility, adherence, and operational insights of ABLE
Source: PLOS Digit Health. 2026 Sep 25;5(9):e0001691. doi: 10.1371/journal.pdig.0001691 (PMC13614584; doi:10.1371/journal.pdig.0001691)
Supplement: Supplementary file 5 [file pdig.0001691.s005.docx]

**S4 Fig. Kaplan-Meier curve for time-to-event analysis of participant dropout from the study.** Blue refers to the AKG intervention group and orange refers to the placebo group at each study visit.
