# Supplementary material for: Optimizing accelerometer implementation in a gerotherapeutic trial: Feasibility, adherence, and operational insights of ABLE
Source: PLOS Digit Health. 2026 Sep 25;5(9):e0001691. doi: 10.1371/journal.pdig.0001691 (PMC13614584; doi:10.1371/journal.pdig.0001691)
Supplement: Supplementary file 6 [file pdig.0001691.s006.docx]

**Changes to – manuscript number PDIG-D-26-00240 entitled ‘Optimizing accelerometer implementation in a gerotherapeutic trial: Feasibility, adherence, and operational insights of ABLE’**

We thank the editor and reviewers for the effort they have put into providing constructive comments. We have modified the manuscript according to the editor’s suggestions, which were very helpful and improved the manuscript considerably. The questions and reviewers’ comments are given in ‘**bold font’**, our responses are given in ‘normal font’, and the textual revisions are given in ‘*italic font*’. We highlighted the textual revisions in the manuscript using ‘red and underlined’ font.

**Editor:**

**Comment 1: Dear authors, thank you for submitting a paper on a timely topic - the use of technology to support the elderly.**

Author response: Thank you for your appreciation of our work and for recognizing the timeliness of research on technology-supported gerotherapeutic studies.

**Comment 2: I found it intriguing that there was a difference in male and female adherence. Unfortunately, despite your very detailed results and data, I did not find any hypothesis on the difference. I also did not see a section labeled conclusion. An explanation on what might have caused this gender difference in behavior and what may be done to decrease this gap could be the contribution of this paper to the literature.**

Author response: Thank you for the insightful suggestions. We note that the observed sex-related association was with dropout risk rather than accelerometer wear adherence. We have expanded the Discussion to interpret the observed sex-related difference in dropout risk more clearly and cautiously. In our adjusted Cox model, being male was associated with lower dropout risk, while the main recorded reason for withdrawal was work/schedule-related conflict. We therefore propose that differential work–family demands, caregiving responsibilities, and perceived study burden may be plausible contributors to the higher dropout risk observed among female participants. However, because caregiving responsibilities and perceived burden were not directly measured, we have framed these as hypotheses for future study rather than causal explanations. We have also added a “Conclusion” heading to clearly identify the concluding section.

*Changes to manuscript, page 21–22: The primary reasons for withdrawal were dominated by work/schedule-related conflicts, a common challenge in studies with this demographic, followed by lost to follow-up. Another key finding was the association between device wear time and study dropout risk. Higher wearing time and being male were significantly associated with lower odds of dropout. This suggests that adherence to wearing the device could serve as a proxy for overall study engagement, commitment, or perhaps perceived benefit, which in turn influences participant retention. This finding warrants further exploration, potentially relating to differing motivations, health perceptions, or tolerance for study burden between sexes in this cohort.^48^ One plausible explanation is that some female participants may have experienced higher perceived study burden because of competing work, family, and caregiving responsibilities, which have been identified as barriers to clinical trial participation in prior research.^48,49^ However, the present study did not measure caregiving responsibilities or perceived study burden. These mechanisms should, therefore, be considered hypothesis-generating and can be assessed in future gerotherapeutic trials.*

*Changes to manuscript, pages 22–23:* *The consistent individual adherence patterns across visits observed suggest that early compliance may lead to later device adherence, allowing for targeted support. The combination of these strategies may have contributed to a higher proportion of participants providing sufficient valid days for PA analysis. Given that male sex was associated with lower dropout risk in this analysis, future studies should consider whether additional retention support is needed for female participants or other groups with competing work or caregiving demands.^51^ Such support should be designed to reduce burden rather than to assume non-adherence, and future trials should directly measure potential mediators such as caregiving responsibilities, work constraints, perceived burden, and motivation.^52^*

*Changes to manuscript, page 23:*

*[heading added]* ***Conclusion***

*The current findings underscore that accelerometer integration is feasible and can yield high-quality data in middle-aged adults in a gerotherapeutic interventional trial. Future gerotherapeutic trials should consider study designs for robust participant support and device management. The operational insights offer valuable guidance for optimizing the design and execution of future aging research incorporating wearable technology.*

**Comment 3: I also do not see any of the figures although I will check again if there is a separate file for that. I would expect a figure showing the flow from the first 120 clients gradually diminishing because of the exclusions.**

Author response: Thank you for noting this. The figures were submitted as separate Figure documents entitled “CONSORT_2025_flow_diagram.pdf” and “PA adherence & feasibility_Figures 2&3.docx”; however, it appears that the compiled submission PDF may not have displayed them correctly. We have now uploaded the figures individually in .tif format in accordance with the journal requirements. Figure 1 is the CONSORT-style participant flow diagram and shows participant progression from screening/randomization through follow-up and inclusion in the accelerometer analyses.

*Changes to manuscript: Not applicable.*

**Comment 4: Finally I would expect a simple recommendation to help in improving the adherence esp for the males...**

Author response: Thank you for the helpful suggestion. Because being male was associated with lower dropout risk in our study, we have clarified the Discussion to focus on strategies for participants who may be at higher risk of dropout, including female participants. We agree that the manuscript should provide practical recommendations. We have, therefore, added specific strategies for future trials, including flexible visit scheduling, early identification of participants with lower wear time, proactive check-ins, accessible troubleshooting, alternative strap options to address comfort and hygiene concerns, simplified device return procedures, and consideration of devices with periodic or near real-time data transfer for longer studies. These recommendations are intended to reduce logistical, comfort-related, and perceived participation burden while maintaining high-quality accelerometer data capture.

*Changes to manuscript, pages 22–23: This study provides several actionable insights for future trials, particularly gerotherapeutic trials, incorporating accelerometers in middle-aged community-dwelling adults, such as participant onboarding and support, device management and data retrieval, optimizing wear protocols, and data quality and quality control,^40^ all of which aid in achieving a high data yield. Clear instructions, accessible troubleshooting (e.g., WhatsApp support), and managing expectations regarding device wear (including aesthetics and comfort) are crucial. Proactive strap replacement or material options can mitigate comfort concerns. Strategies to minimize data loss from lost devices are also needed. While mail-back is logistically simple, future studies could explore devices with periodic or near real-time data syncing capabilities, especially for longer wear periods or more critical data endpoints.^50^ A standard seven-day wear protocol^33^ post-visit appears manageable for this population. Moreover, defining clear non-wear criteria, valid day definitions, and monitoring wear time variability can ensure data reliability. The consistent individual adherence patterns across visits observed suggest that early compliance may lead to later device adherence, allowing for targeted support. The combination of these strategies may have contributed to a higher proportion of participants providing sufficient valid days for PA analysis. Given that being male was associated with lower dropout risk in this analysis, future studies should consider whether additional retention support is needed for female participants or other groups with competing work or caregiving demands.^51^ Such support should be designed to reduce burden rather than to assume non-adherence, and future trials should directly measure potential mediators such as caregiving responsibilities, work constraints, perceived burden, and motivation.^52^*

**Reviewer(s):**

**No comments in Decision Letter and no files attached to the decision notification email.**
