# Supplementary material for: Optimizing accelerometer implementation in a gerotherapeutic trial: Feasibility, adherence, and operational insights of ABLE
Source: PLOS Digit Health. 2026 Sep 25;5(9):e0001691. doi: 10.1371/journal.pdig.0001691 (PMC13614584; doi:10.1371/journal.pdig.0001691)
Supplement: Supplementary file 7 [file pdig.0001691.s007.docx]

**Changes to – manuscript number PDIG-D-26-00240R1 entitled ‘Optimizing accelerometer implementation in a gerotherapeutic trial: Feasibility, adherence, and operational insights of ABLE’**

We thank the editor and reviewer for the effort they have put into providing constructive comments. We have modified the manuscript according to the editor’s suggestions, which were very helpful and improved the manuscript considerably. The questions and reviewers’ comments are given in ‘**bold font’**, our responses are given in ‘normal font’, and the textual revisions are given in ‘*italic font*’. We highlighted the textual revisions in the manuscript using ‘red and underlined’ font.

**Editor:**

**No comments in Decision Letter and no files attached to the decision notification email.**

**Reviewer:**

**Comment 1: The revised text is generally clear, but a few language and formatting issues remain, such as “This aim,” “ethnical,” “Date is presented,” and “gerotherapuetic/gerotherapueutic.”**

Author response: Thank you for noting these language and formatting issues. We have reviewed the manuscript and corrected the identified typographical and grammatical errors, including “This aim,” “ethnical,” “Date is presented,” and “gerotherapuetic/gerotherapueutic.” We also conducted an additional review of the manuscript to correct similar errors and improve consistency in terminology.

*Changes to manuscript, page 4: The aim of this study is to investigate the operational aspects of deploying accelerometers, participant adherence to the accelerometer protocol and supplement use, and associations between accelerometer wear adherence and study dropout in the Alpha-ketoglutarate (AKG) supplementation and BiologicaL agE in middle-aged adults (ABLE) study.^14^*

*Changes to manuscript, pages 5 and 26 : The study was approved by the National University of Singapore Institutional Review Board (NUS-IRB Ref: 2021-946) with all ethical guidelines adhered to under the Declaration of Helsinki.^15^*

*Changes to manuscript, pages 14 and 17: Date are presented as mean ± standard deviation (SD) or median [interquartile range (IQR)] unless otherwise stated.*

*Changes to manuscript, page 20: Table 5. The association between device wear time and dropout status in middle-aged, healthy adults in a gerotherapeutic trial (N = 119).*

**Comment 2: Handling of missing sleep/wake times and diary-based corrections is not fully reproducible. Author should add brief decision rules or quality-control details.**

Author response: Thank you for this helpful comment. We have revised the Methods section to provide clearer decision rules for handling missing sleep/wake times and diary-based corrections. Specifically, we now describe how missing sleep/wake times were estimated using accelerometer movement patterns and available physical activity diary information, how diary-recorded monitor removal periods were cross-checked against ActiGraph-detected non-wear periods, and how discrepancies were handled during quality control. These additions were made to improve the reproducibility of the accelerometer data processing workflow.

*Changes to manuscript, page 8: The participants were asked to record daily in the electronic PA diary the exercises that they engage in and their monitor removal times (and why it was removed, e.g., shower, swimming, etc.). When sleep/wake times were missing, study staff estimated sleep and wake periods using the accelerometer movement pattern together with available diary entries for adjacent days. Diary-recorded device removal periods were cross-checked against ActiGraph-detected non-wear periods. Where diary entries and accelerometer signals were discrepant, accelerometer-derived non-wear detection was prioritized for wear-time calculations.*

**Comment 3: Cox model needs clearer reporting on the time-varying wear variables, clustering unit, proportional-hazards checks, and how wear data were aligned before dropout.**

Author response: Thank you for this comment. We have clarified the Cox model reporting in the Methods, including the visit-level time-varying structure of device wear variables, clustering by participant, and alignment of wear data with subsequent dropout intervals. We also assessed the proportional hazards assumption using Schoenfeld residuals. This assessment indicated no evidence of non-proportionality for device wear time, sex, or intervention group, although the global test suggested possible non-proportionality driven by age. Because age was included as an adjustment covariate rather than a primary exposure, we therefore conducted a sensitivity analysis excluding age from the model, and the associations for device wear time and sex remained consistent. We have revised the Methods and Results accordingly.

*Changes to manuscript, pages 11–12: Time-to-dropout was analysed using Kaplan-Meier time-to-event analysis and Cox proportional hazards models to identify factors associated with dropout risk over the course of the study. Study visit was used as the time scale, with dropout defined as the first missed follow-up visit or formal withdrawal after baseline. Device wear time and device wear adherence were modelled as visit-level time-varying covariates. For each risk interval, wear data from the preceding monitoring period were aligned with subsequent dropout status, such as accelerometer wear after a completed visit was used to model dropout before the next scheduled visit. Cox models used cluster-robust standard errors clustered by participant to account for repeated visit-level observations. Candidate time-varying covariates, including device wear time and device wear adherence, were screened in univariate models. Because device wear time and wear adherence were highly collinear, the variable with the superior concordance index in univariate screening was chosen for multivariable analysis. The adjusted Cox model included device wear time, age, sex, and intervention or placebo group covariates and accounted for the time to dropout. The proportional hazards assumption was assessed using Schoenfeld residuals. Where evidence of non-proportionality was detected for adjustment covariates, sensitivity analyses were conducted to evaluate whether the main associations for device wear time and sex were robust.*

*Changes to manuscript, pages 18–19: One minute higher in wearing time was associated with a 0.2% lower hazard risk of dropping out (HR: 0.998; 95% CI: 0.997–0.999; p < 0.001) as well as being male (HR: 0.208; 95% CI: 0.065–0.663; p = 0.008) in the adjusted multivariate Cox model. Schoenfeld residual testing showed no evidence of non-proportionality for device wear time, sex, or intervention group in the primary adjusted model, although age showed evidence of non-proportionality and the global test was significant. In a sensitivity model excluding age, the proportional hazards assumption was met globally and for all included covariates. Device wear time and sex remained associated with dropout risk, with estimates similar to the primary adjusted model (wear time HR: 0.998, 95% CI: 0.997–0.999, p < 0.001; sex HR: 0.198, 95% CI: 0.063–0.624, p = 0.006).*

**Comment 4: The manuscript would benefit from citing "Smart sensors for Hospital 4.0/5.0: an introduction". This work is relevant because it discusses smart sensors, continuous health monitoring, and real-time healthcare data collection, which directly support the manuscript’s focus on wearable accelerometers and digital phenotyping. It can be added in the Intro or Discussion sec.**

Author response: Thank you for this suggestion. We reviewed the suggested chapter and have added it to the Introduction to support the broader context of smart sensors and continuous monitoring. Because the chapter focuses primarily on hospital-based smart sensor systems, we have retained more directly relevant wearable and free-living accelerometry references for the study-specific rationale and operational discussion in community-dwelling adults.

*Changes to manuscript, pages 4: Digital phenotyping tools (e.g., smart sensors, accelerometers, and wearable devices) enable continuous, objective measurement of both device wear adherence and free-living activity.^5,6^ Moreover, by capturing candidate digital biomarkers of aging, these devices can help reveal early signs of intervention responsiveness that traditional endpoints miss.^7^*

**Comment 5: Reported 17% dropout rate appears difficult to reconcile with the stated 92% study completion probability and the flow diagram counts. Author should reverify this.**

Author response: Thank you for identifying this inconsistency. We rechecked the Kaplan–Meier analysis and confirmed that the value of 0.92 referred to the log-rank test *p*-value comparing time-to-dropout between the AKG and placebo groups, not the study completion probability. We have corrected the Results text accordingly. The overall dropout rate was 17% (20/120), corresponding to an overall completion proportion of 83% (100/120), with similar completion proportions in the AKG and placebo groups. We have also updated Supplementary Figure 4 by revising the y-axis label to “Probability of remaining in the study” to more accurately reflect the Kaplan–Meier curve.

*Changes to manuscript, page 18: The overall study dropout rate was 17% (20 out of 120 participants dropped out after randomization to the AKG or placebo groups). The primary reasons for withdrawal were work/schedule-related conflicts (n = 10), followed by lost to follow-up or uncontactable (n = 4). Other reasons included study product-related issues (n = 2) and protocol non-compliance (n = 2), as detailed in* ***Table 4****. A time-to-event analysis of study dropout showed no significant difference between the groups (log-rank p = 0.92), consistent with ~83% completion probability in both groups (****Supplementary Figure 4****).*

*Changes to Supplementary File, page 6:*

***Figure 4. Kaplan-Meier curve for time-to-event analysis of participant dropout from the study.*** *Blue refers to the AKG intervention group and orange refers to the placebo group at each study visit.*
