# Supplementary material for: Optimizing accelerometer implementation in a gerotherapeutic trial: Feasibility, adherence, and operational insights of ABLE
Source: PLOS Digit Health. 2026 Sep 25;5(9):e0001691. doi: 10.1371/journal.pdig.0001691 (PMC13614584; doi:10.1371/journal.pdig.0001691)
Supplement: Peer review history

# Editor report: Decision Letter 0

**DOI:** 10.1371/journal.pdig.0001691.r001

**Authors:** Lawrence D Hayes, Alvin Valeriano de Borja Marcelo

19 May 2026

Response to Reviewers Revised Manuscript with Track Changes Manuscript **Journal Requirements:**

1.  In the online submission form, you indicated that “The dataset and code used in this study is available upon reasonable request to the corresponding author.”.

All PLOS journals now require all data underlying the findings described in their manuscript to be freely available to other researchers, either

1. In a public repository,

2. Within the manuscript itself, or

3. Uploaded as supplementary information.

This policy applies to all data except where public deposition would breach compliance with the protocol approved by your research ethics board. If your data cannot be made publicly available for ethical or legal reasons (e.g., public availability would compromise patient privacy), please explain your reasons by return email and your exemption request will be escalated to the editor for approval. Your exemption request will be handled independently and will not hold up the peer review process, but will need to be resolved should your manuscript be accepted for publication. One of the Editorial team will then be in touch if there are any issues.

2. Please ensure that your Ethics Statement is available in its entirety at the beginning of your Methods section, under a subheading 'Ethics Statement'.

3. Please provide separate figure files in .tif or .eps format.

For more information about figure files please see our guidelines:

https://journals.plos.org/digitalhealth/s/figures

https://journals.plos.org/digitalhealth/s/figures#loc-file-requirements

4. We do not publish any copyright or trademark symbols that usually accompany proprietary names, eg (R), (C), or TM  (e.g. next to drug or reagent names). Please remove all instances of trademark/copyright symbols throughout the text, including ActiGraph wGT3X-BT® and CentrePoint®.

5. We have noticed that you have uploaded Supporting Information files, but you have not included a list of legends. Please add a full list of legends for your Supporting Information files after the references list.

**Additional Editor Comments (if provided):**

**Reviewers' Comments:** **Figure resubmission:**

**Reproducibility:**

To enhance the reproducibility of your results, we recommend that authors of applicable studies deposit laboratory protocols in protocols.io, where a protocol can be assigned its own identifier (DOI) such that it can be cited independently in the future. Additionally, PLOS ONE offers an option to publish peer-reviewed clinical study protocols. Read more information on sharing protocols at https://plos.org/protocols?utm_medium=editorial-email&utm_source=authorletters&utm_campaign=protocols

# Author comment: Author response to Decision Letter 1

**DOI:** 10.1371/journal.pdig.0001691.r002

6 Jun 2026

Attachment Submitted filename: Response to Reviewers_PLOS DH.docx

# Peer review: Decision Letter 1

**DOI:** 10.1371/journal.pdig.0001691.r003

**Authors:** Lawrence D Hayes

23 Jul 2026

Response to ReviewersRevised Manuscript with Track ChangesManuscript**Journal Requirements:** **Additional Editor Comments (if provided):** **Reviewers' Comments:**

**Comments to the Author**

Reviewer #1: All comments have been addressed

**********

publication criteria?

Reviewer #1: Yes

**********

3. Has the statistical analysis been performed appropriately and rigorously?

Reviewer #1: Yes

**********

4. Have the authors made all data underlying the findings in their manuscript fully available (please refer to the Data Availability Statement at the start of the manuscript PDF file)?

The PLOS Data policy

Reviewer #1: Yes

**********

5. Is the manuscript presented in an intelligible fashion and written in standard English?

Reviewer #1: Yes

**********

**Review Comments to the Author**

Reviewer #1: 1. The revised text is generally clear, but a few language and formatting issues remain, such as “This aim,” “ethnical,” “Date is presented,” and “gerotherapuetic/gerotherapueutic.”

2. Handling of missing sleep/wake times and diary-based corrections is not fully reproducible. Author should add brief decision rules or quality-control details.

3. Cox model needs clearer reporting on the time-varying wear variables, clustering unit, proportional-hazards checks, and how wear data were aligned before dropout.

4. The manuscript would benefit from citing "Smart sensors for Hospital 4.0/5.0: an introduction". This work is relevant because it discusses smart sensors, continuous health monitoring, and real-time healthcare data collection, which directly support the manuscript’s focus on wearable accelerometers and digital phenotyping. It can be added in the Intro or Discussion sec.

5. Reported 17% dropout rate appears difficult to reconcile with the stated 92% study completion probability and the flow diagram counts. Author should reverify this.

**********

what does this mean?). If published, this will include your full peer review and any attached files.

**Do you want your identity to be public for this peer review?**  If you choose “no”, your identity will remain anonymous but your review may still be made public.

For information about this choice, including consent withdrawal, please see our Privacy Policy

Reviewer #1: No

**********

**Figure resubmission:**

**Reproducibility:**

To enhance the reproducibility of your results, we recommend that authors of applicable studies deposit laboratory protocols in protocols.io, where a protocol can be assigned its own identifier (DOI) such that it can be cited independently in the future. Additionally, PLOS ONE offers an option to publish peer-reviewed clinical study protocols. Read more information on sharing protocols at https://plos.org/protocols?utm_medium=editorial-email&utm_source=authorletters&utm_campaign=protocols

# Author comment: Author response to Decision Letter 2

**DOI:** 10.1371/journal.pdig.0001691.r004

1 Aug 2026

Attachment Submitted filename: Response_to_Reviewers_PLOS_DH_auresp_2.docx

# Peer review: Decision Letter 2

**DOI:** 10.1371/journal.pdig.0001691.r005

**Authors:** Lawrence D Hayes

10 Aug 2026

Optimizing accelerometer implementation in a gerotherapeutic trial: Feasibility, adherence, and operational insights of ABLE

PDIG-D-26-00240R2

Dear Prof. Dr. Dr. Maier,

We are pleased to inform you that your manuscript 'Optimizing accelerometer implementation in a gerotherapeutic trial: Feasibility, adherence, and operational insights of ABLE' has been provisionally accepted for publication in PLOS Digital Health.

Before your manuscript can be formally accepted you will need to complete some formatting changes, which you will receive in a follow-up email from a member of our team.

Please note that your manuscript will not be scheduled for publication until you have made the required changes, so a swift response is appreciated.

IMPORTANT: The editorial review process is now complete. PLOS will only permit corrections to spelling, formatting or significant scientific errors from this point onwards. Requests for major changes, or any which affect the scientific understanding of your work, will cause delays to the publication date of your manuscript.

If your institution or institutions have a press office, please notify them about your upcoming paper to help maximize its impact. If they'll be preparing press materials, please inform our press team as soon as possible -- no later than 48 hours after receiving the formal acceptance. Your manuscript will remain under strict press embargo until 2 pm Eastern Time on the date of publication. For more information, please contact digitalhealth@plos.org.

Thank you again for supporting Open Access publishing; we are looking forward to publishing your work in PLOS Digital Health.

Best regards,

Lawrence D Hayes, PhD

Section Editor

PLOS Digital Health

***********************************************************

**Additional Editor Comments (if provided):**

**Reviewer Comments (if any, and for reference):**

Reviewer's Responses to Questions

**Comments to the Author**

Reviewer #1: All comments have been addressed

**********

publication criteria?

Reviewer #1: Yes

**********

3. Has the statistical analysis been performed appropriately and rigorously?

Reviewer #1: Yes

**********

4. Have the authors made all data underlying the findings in their manuscript fully available (please refer to the Data Availability Statement at the start of the manuscript PDF file)?

The PLOS Data policy

Reviewer #1: Yes

**********

5. Is the manuscript presented in an intelligible fashion and written in standard English?

Reviewer #1: Yes

**********

Reviewer #1: Changes made in response to Reviewer comments look good and have substantially addressed the previous concerns. In particular, authors have clarified the accelerometer data-processing rules, provided more detail on the time-varying Cox regression and proportional-hazards assessment, corrected the interpretation of the dropout analysis.

**********

what does this mean?). If published, this will include your full peer review and any attached files.

**Do you want your identity to be public for this peer review?**  If you choose “no”, your identity will remain anonymous but your review may still be made public.

For information about this choice, including consent withdrawal, please see our Privacy Policy

Reviewer #1: No

**********
